# Fluorescence-coded DNA Nanostructure Probe System to Enable Discrimination of Tumor Heterogeneity via a Screening of Dual Intracellular microRNA Signatures *in situ*

**DOI:** 10.1038/s41598-017-13456-3

**Published:** 2017-10-18

**Authors:** Seung Won Shin, Byoung Sang Lee, Kisuk Yang, Lunjakorn Amornkitbamrung, Min Su Jang, Bo Mi Ku, Seung-Woo Cho, Jung Heon Lee, Hojae Bae, Byung-Keun Oh, Myung-Ju Ahn, Yong Taik Lim, Soong Ho Um

**Affiliations:** 1 School of Chemical Engineering, Sungkyunkwan University, Suwon, Gyeonggi-do South Korea; 2 SKKU Advanced Institute of Nanotechnology (SAINT), Sungkyunkwan University, Suwon, Gyeonggi-do South Korea; 3 School of Advanced Materials Science and Engineering, Sungkyunkwan University, Suwon, Gyeonggi-do South Korea; 4 Department of Biotechnology, Yonsei University, Seoul, South Korea; 5 Samsung Biomedical Research Institute, Samsung Medical Center, Sungkyunkwan University School of Medicine, Seoul, South Korea; 6 Division of Hematology-Oncology, Department of Medicine, Samsung Medical Center, Sungkyunkwan University School of Medicine, Seoul, South Korea; 7 College of Animal Bioscience and Technology, Department of Bioindustrial Technologies, Konkuk University, Seoul, South Korea; 8 Department of Chemical & Biomolecular Engineering, Sogang University, Seoul, South Korea

## Abstract

Since the delivery kinetics of different cell types are different, the signal from the target cell is greatly affected by the noise signal of the diagnostic system. This is a major obstacle hindering the practical application of intracellular diagnostic systems, such as tumor heterogeneity. To address these issues, here we present a microRNA detection platform using fluorescence-encoded nanostructured DNA-based probes. The nanostructured DNA was designed to include molecular beacons for detecting cytosolic microRNA as well as additional fluorophores. When the intracellular diagnostic system is delivered, fluorescence signals are generated by the molecular beacons, depending on the concentration of the target microRNA. The fluorescence signals are then normalized to the intensity of the additional fluorophore. Through this simple calculation, the concentration of intracellular microRNA can be determined without interference from the diagnosis system itself. And also it enabled discrimination of microRNA expression heterogeneity in five different breast cancer cell lines.

## Introduction

Developments in cell biology and molecular cell biology have revealed the metabolic and signaling pathways occurring in various biological systems^1,2^. This achievement has shed light on the underlying causes of disease and facilitated detection of biomarkers for the diagnosis of different diseases. For decades, intense efforts have focused on the identification of effective biomarkers for cancer therapeutics and the elucidation of their molecular mechanisms^3–5^. Engineering technologies have also been developed for detecting specific biomarkers from biological samples and for suggesting appropriate treatment^6–10^. At present, multiple diagnostic strategies are able to detect cancer-specific proteins or nucleic acid molecules, even at the femtomolar level, in various types of biological samples.

However, many engineering technologies have not yet reached practical applications for medical purposes^11–13^. Several hurdles must be overcome for the commercialization of new medical technologies, such as achieving acceptable reproducibility and biocompatibility. More basically, for practical medical applications of a given technology, it is important to optimize and refine the technology to suit the intended purpose and methodology. For intracellular diagnosis, various strategies have been developed for the effective detection of biomarkers in single cells^14–16^. In particular, microRNAs (miRNAs) have recently emerged as important molecules and diagnostic techniques for cancer-specific miRNA detection have been reported^17–20^. Effective miRNA-detecting tools, such as molecular beacons, have been proposed; various techniques have also been developed for effective delivery of diagnostic systems into target cells. However, additional factors must be considered to use intracellular diagnostic techniques for practical medical applications.

Even cancer cells that originate from the same tumor site show genetic and phenotypic diversity, known as tumor heterogeneity^21–23^. Progressive cancer has a strong defense system against most developed clinical treatments and can overcome the treatment with tolerance due to the genetic diversity of the cancer cells. Also, multiple cell types are obtained together with the cancer cells of interest when samples are taken from a patient, requiring that intracellular diagnostic strategies incorporate comparisons between different cell types. The most fundamental hurdle for comparing different cell types is that internalization rates of diagnostic systems vary widely depending on the cell type and delivery conditions^24,25^. For example, the SK-BR-3 cell line has significantly faster uptake of certain nanoparticles than the MDA-MB-231 cell line, even though both cell lines are breast cancer-derived. This difference introduces unanticipated background signal from each cell type, making it impossible to directly discriminate the amounts of intracellular biomarkers within different cell types.

Here we address these issues by reporting a novel nanostructured DNA-based probing system for the direct comparison of intracellular miRNAs in different cell lines. The probe is composed of three different compartments: 1) nanostructured DNA, which was designed for quantitative detection of multiplexed miRNAs without interference of cell-specific uptake kinetics; 2) a core silica nanoparticle platform for signal enhancement; and 3) a positively charged lipid layer that possesses high cellular transport efficiency. In addition, the silica nanoparticle and the lipid layer were designed to contribute protection of the nanostructured DNA from DNA-degrading enzymes. Since a plenty of DNA-degrading enzymes exist in the physiological environment, it is important to protect the unique structure of nanostructured DNA to maintain proper functioning^26^. It is known that DNase-induced degradation is efficiently prevented by attaching DNA to the surface of nanoparticles^27–29^. Also, the lipid layer can effectively inhibit the degradation of DNA by blocking access of various DNA-degrading enzymes^30^.

It is known from previous studies that nanostructured DNAs can be applied in wide-range of biomedical fields through their unique properties^31–34^. For instance, precisely designed nanostructured DNAs were applied to drug delivery carrier, nanorobot, etc^35^. Recently, it was also revealed that nanostructured DNAs are capable of cellular internalization with high efficiency^36,37^. In this study, the nanostructured DNA was designed to contain molecular beacons and additional fluorophores for target miRNA detection and signal normalization, respectively. When the molecular beacon precisely hybridizes to intracellular miRNAs, a specific fluorescent signal is recovered. In contrast, the fluorescence signal generated by the additional fluorophore is emitted according to the amount of the nanostructured DNA transferred into the cell. By normalizing the two different fluorescence signals, the concentration of target intracellular miRNA can be compared without knowing the precise amount of delivered nanostructured DNA-based probe. To evaluate the diagnostic potential of this system, five different breast cancer cell lines were chosen as model cell lines for miRNA detection due to their well-characterized genetic profiles and clinical significance^38^. The target miRNAs miR-21 and miR-22 were used. miR-21 was selected as a representative oncogenic miRNA since it is overexpressed in many cell lines^39,40^. Moreover, miR-21 has been found to be associated with several genes whose expression correlates with cancer metastasis. These genes include tumor suppressor tropomyosin 1 (TPM1), phosphatase and tensin homolog (PTEN), and B-cell lymphoma 2 (BCL2). miR-22 has also been shown to be associated with the epithelial-mesenchymal transition (EMT), which enhances cancer invasiveness, and to promote metastasis^41^. This study first characterized the nanostructured DNAs, which included analyzing their structural stability, target detection sensitivity, and selectivity. Next, the silica core platform with a lipid fusion layer was optimized for efficient cellular delivery and signal enhancement. Finally, the multiplexed miRNA levels were compared in five different breast cancer cell lines.

## Results

### Differences in the intracellular diagnostic system delivery by cell lines

**Figure 1:**
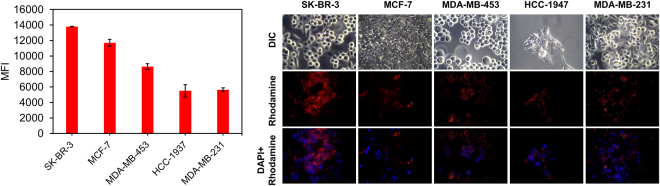
Flow cytometry and fluorescence microscopy images of DOTAP-coated silica nanoparticle-treated breast cancer cell lines. For the treatment, 500 μg of rhodamine b-labeled silica nanoparticles was incubated for 2 hours with the cell lines, after which cells were harvested for analysis. Although the treatment conditions were the same, each cell line showed significantly different fluorescent signals.

First, five different breast cancer cell lines (SK-BR-3, MCF-7, HCC-1947, MDA-MB-231, and MDA-MB-453) were treated with the same amount of dioleoyl-trimethylammonium-propane (DOTAP)-coated silica nanoparticles to determine whether the nanoparticles had different cellular uptake kinetics in the cell lines. The silica nanoparticles were 109.0 ± 2.3 nm in diameter. For the fluorescence microscopy imaging and flow cytometry analysis, silica nanoparticles were labeled with rhodamine b. After 2 hours of incubation, cells were harvested and analyzed. As shown in Fig. 1, each cell line showed different intracellular particle uptake. Of particular note, the mean fluorescence intensity (MFI) of SK-BR-3 cells was almost 2.5 times higher than that of HCC-1947 cells. This result demonstrates differences in intracellular delivery, raising the possibility of inaccurate interpretation.

### Conformation analysis of fc-DNA and afc-DNA

**Figure 2:**
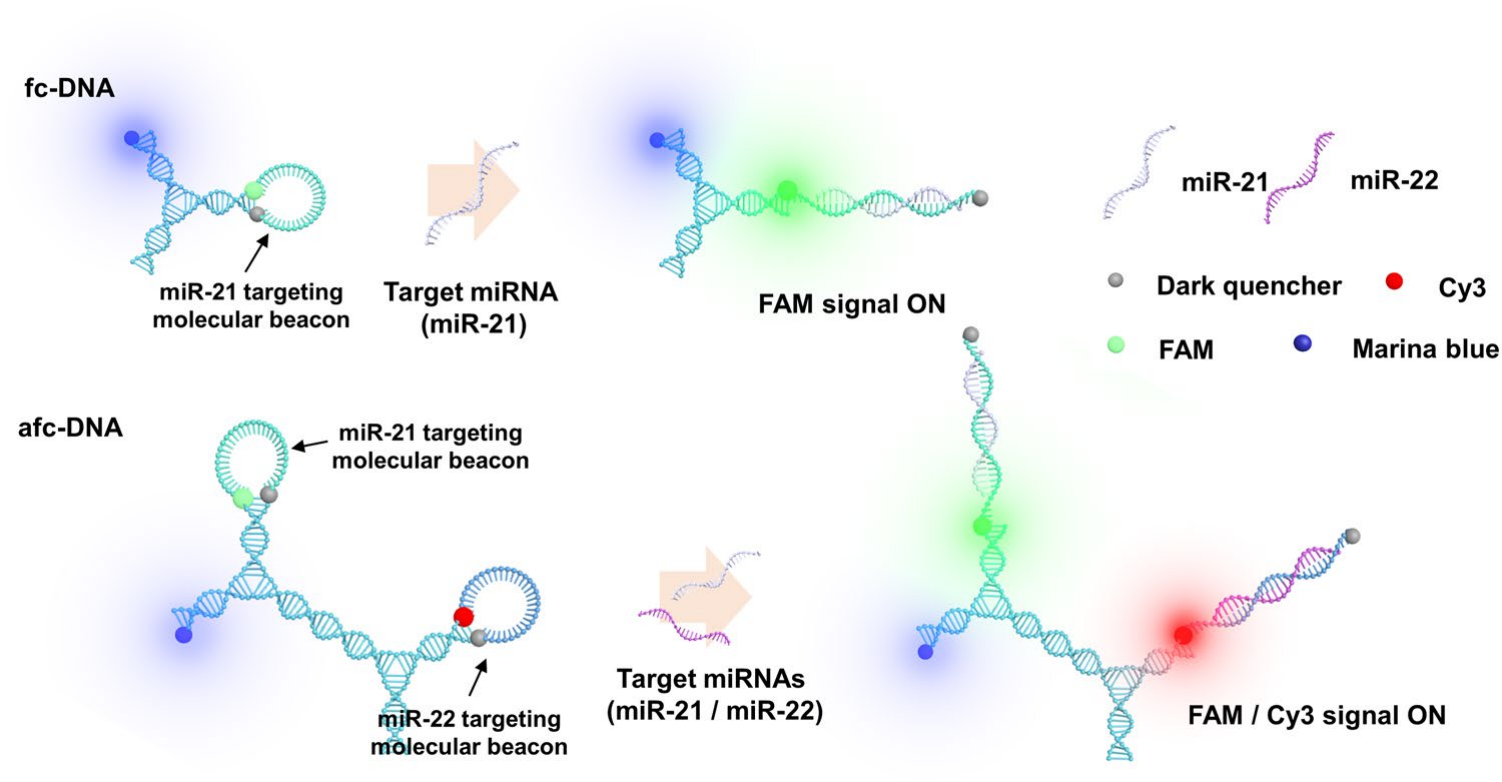
Schematic drawing of the miRNA detection mechanism of fc-DNA and afc-DNA. Fluorescence signal from a molecular beacon was induced by hybridization of the target miRNA strand. fc1- and fc2-DNA targeting different miR-22 and miR-21 were combined by enzymatic ligation to form afc-DNA for multiplexed detection of miRNAs.

As shown in Fig. 2, we assembled a molecular beacon and additional standard fluorophore in a single DNA nanostructure. Due to structural similarity, nanostructured DNA was named fiddler crab-like DNA (fc-DNA), with a large clawed-arm and small arm. With enzymatic cross-linkage of two different fc-DNAs, which were targeting specific miRNAs, precisely assembled fiddler crab-like DNA (afc-DNA) was designed for multiple miRNA detection. A fc-DNA and afc-DNA were built to quantify multiple miRNAs diagnosis. In Supplementary Fig. 1a, the oligonucleotide sequences for nanostructured DNA were noted. fc-DNA was obtained by hybridization of three different oligonucleotides. afc-DNA was synthesized by T4-ligase crosslinking of two different fc-DNAs (fc1-DNA and fc2-DNA). fc1- and fc2-DNA contained a molecular beacon structure that targeted miR-22 and miR-21, respectively. To precisely induce the fc-DNAs ligation, four-base ends, such as AGTC and GACT were selected and added to one of the fc-DNA arms^42^. Formation of fc-DNA and afc-DNA was investigated using gel electrophoresis. As shown in Supplementary Fig. 1b, band locations of fc-DNA and afc-DNA were shifted compared to those of controls (i.e., single oligos), depending on the higher molecular weights and conformational topology.

**Figure 3:**
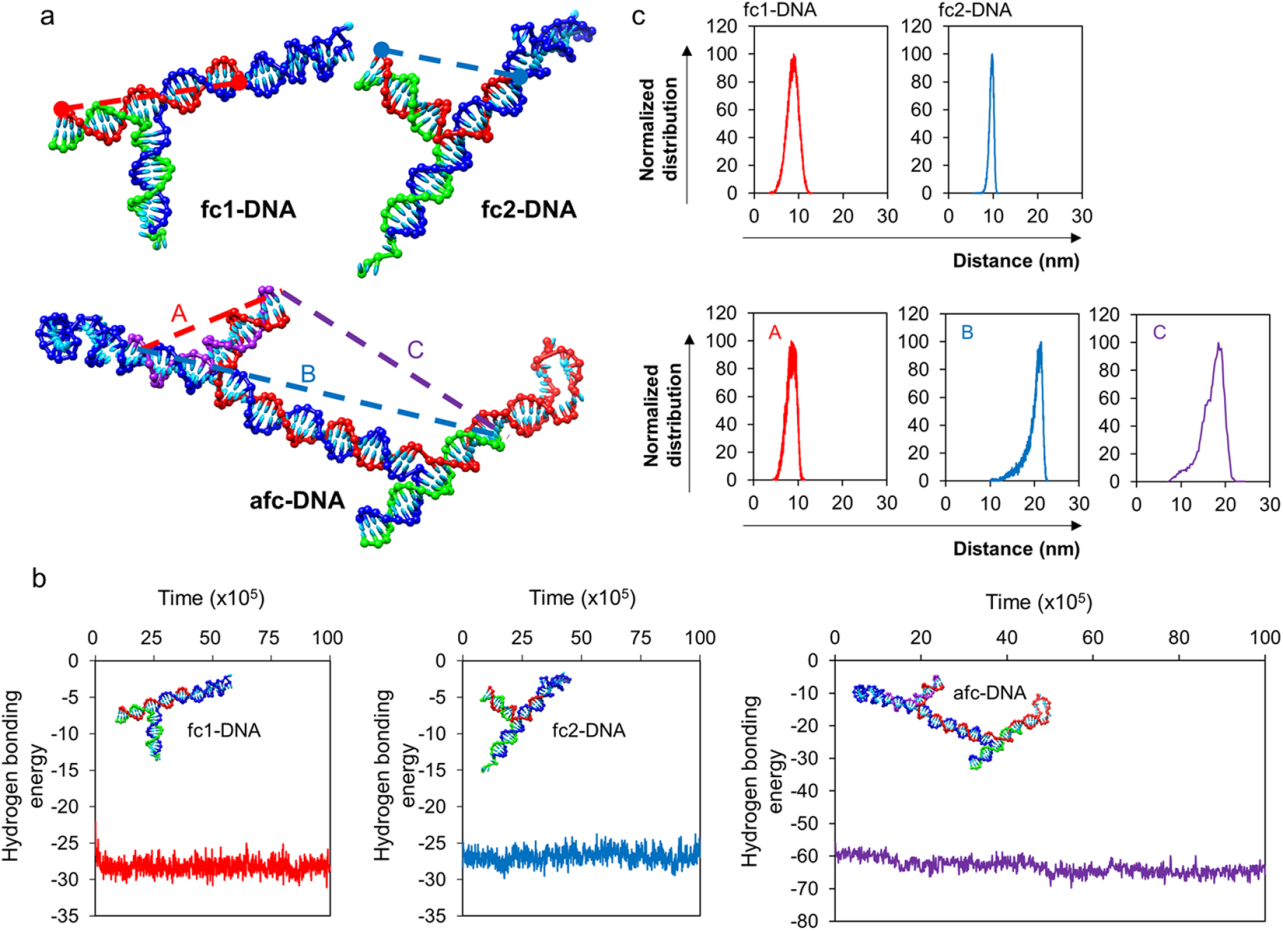
Simulation data of fc-DNAs and afc-DNA. (**a**) Structures of both fc-DNAs and afc-DNA were obtained from oxDNA simulation. (**b**) Hydrogen bonding energy of fc-DNAs and afc-DNA. The hydrogen bond energy of each DNA nanostructure was acquired during the structural analysis step, and all three DNA nanostructures constantly maintained their values. Hydrogen bond energy and time value used in this Figure are in simulation units (SU). The relationship between SU and International System of Units (SI) can be found on the oxDNA program website (http://dna.physic.ox.ac.uk/). (**c**) Normalized distribution of distance in both the fc-DNAs and afc-DNA arms. Distance distributions of afc-DNA (A,B,C) are marked in (**a**). For normalization, the maximum distribution value was reduced to 100.

Since multiple fluorophores were used, it is important to maintain constant distance between fluorophores in order to eliminate errors due to inter-fluorescent interferences. In this study, three different fluorophores were modified to each end of the arm of the nanostructured DNA: Fluorescein amidite (FAM), cyanine 3 (Cy3), and Marina blue. Since the emission spectrum of Marina blue and the excitation spectrum of Cy3 barely overlapped to each other^43^, inter-fluorescent interference would not be occurred. Meanwhile, there is a strong possibility that FAM and Marina blue fluorophores interact to each other, since the spectrum overlapped significantly as well as FAM and Cy3^43^. Therefore, to prevent inter-fluorescent interaction, the distance between the fluorophores should be kept above 10 nm constantly^44^. In this respect, nanostructured DNA is also advantageous by acting as a solid platform of fluorophores. The structures of nanostructured DNAs and distances among fluorophores were predicted with a molecular dynamic simulation method using an oxDNA program (Supporting information Section 1). Simulation data of nanostructured DNA revealed that fc-DNAs and afc-DNA maintained their conformation as designed (Fig. 3a). The thermodynamic stability of the nanostructure was verified by hydrogen bonding energy, as well as visually. During the simulation, hydrogen bonding energy was maintained throughout (Fig. 3b). As shown in Fig. 3c, distances between end points of each arm throughout the entire process were measured, and the distribution of distance was noted for fc1-, fc2-, and afc-DNA. Because the oxDNA program only provides a coarse-grain model simulation for either DNA or RNA, the simulation particles where the fluorophores attached were selected for distance measurement. Distance between the ends of each arm of fc1- and fc2-DNA was counted to be 8.67 ± 0.67 nm and 9.59 ± 0.26 nm, respectively. For afc-DNA, two molecular beacon structures and one standard fluorophore existed, so the distances among the three arms were measured. The distances between the three different arms were 9.45 ± 0.50 nm, 19.77 ± 1.03 nm, and 16.97 ± 1.39 nm, respectively. The three-dimensional structures of the nanostructured DNAs were well established, and the distances among the fluorophores were also maintained during the simulation period.

### Optimization of the thermal annealing process for target detection

The thermal annealing process was a major factor for achieving sensitive target detection. In Supplementary Fig. 2a, fluorescence signals decreased initially when the target strand was treated with fc-DNA at 37 °C. It was assumed that this phenomenon was related to the rate-limiting step of hybridization^45^. The rate of molecular beacon-target hybridization is determined by nucleation of hybridization kinetics. During the nucleation step, bases of the target and the closed stem-looped molecular beacon interact with each other, and the potential energy of complex structures temporarily increases, temporarily forming a stable state. This step dominantly affected the target hybridization kinetics of the molecular beacon. Even though the most stable state occurred when all loop strands hybridized with target strands, the signal from the molecular beacon did not appear immediately. The signal from the fc-DNA increased when the reaction was complete. However, it took at least 120 minutes to recover the initial signal intensity, and target concentration-dependent signal correlation was not clear after 240 minutes. This problem was solved by introducing a thermal annealing process (Supplementary Fig. 2b). When the temperature of target-treated fc-DNA solution was decreased from 37 °C to 4 °C by the program decrement of −1 °C per minute after 1 hour of incubation, a substantial correlation between fluorescence signal intensity and target concentration was achieved.

### Target miRNA diagnosis of fluorescence-coded nanostructured DNAs

**Figure 4:**
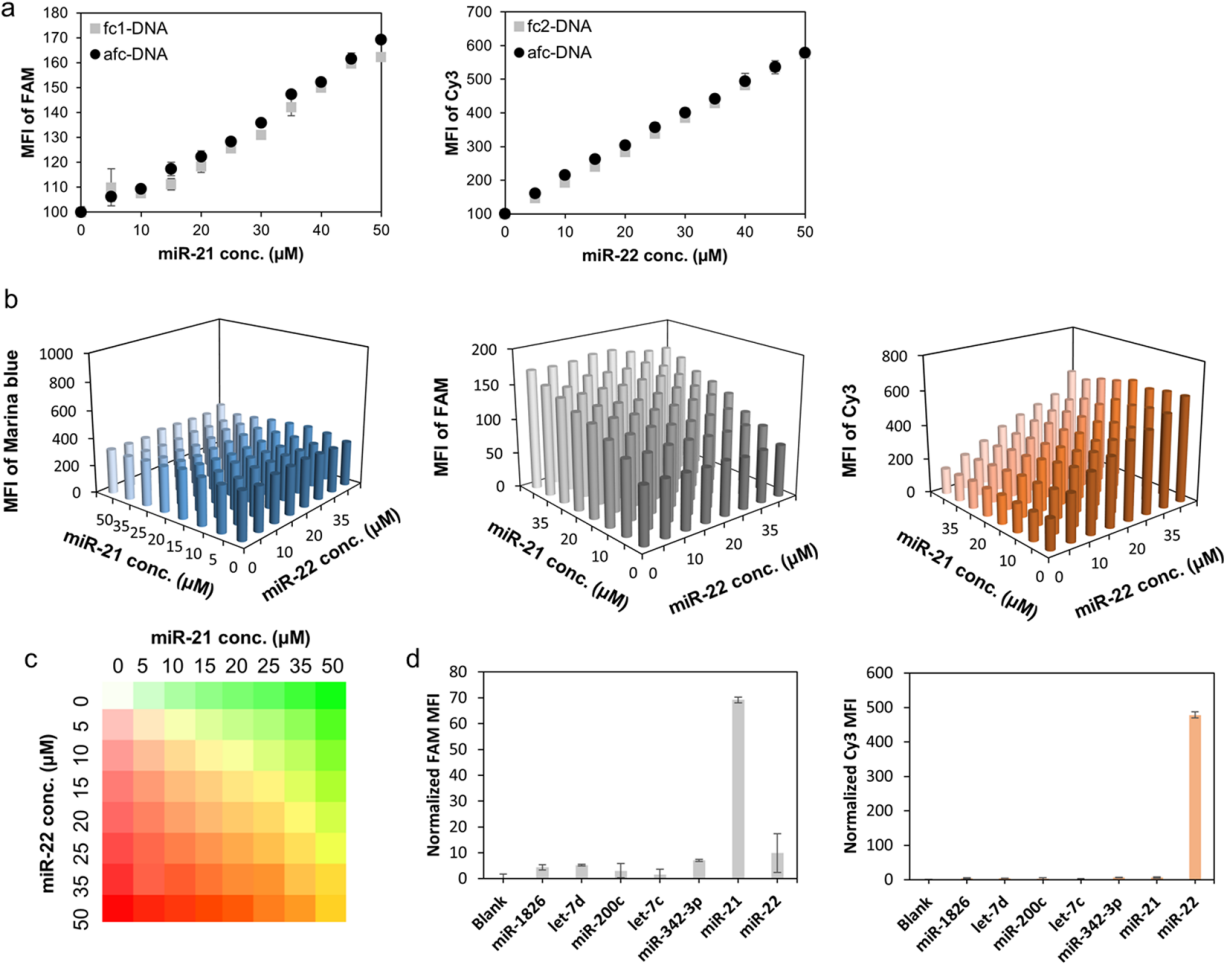
Target detection analysis of fc-DNAs and afc-DNA. (**a**) Target miRNA-dependent signal enhancement in both fc-DNAs and afc-DNA. (**b**) Target-specific fluorescence intensity increments of afc-DNA in miR-21 and miR-22 mixtures. (**c**) Pseudo-color expression of multiplexed detection of afc-DNA according to the miR-21 and miR-22 concentrations. (**d**) Selectivity analysis of afc-DNA for target miRNAs against other breast cancer-related miRNAs.

To test the target detection ability, nanostructured DNA was treated in various concentrations of miR-21 and miR-22. FAM and Cy3 fluorophore-containing molecular beacons were used to detect miR-21 and miR-22, and Marina blue was used as a standard fluorophore. fc-DNA and afc-DNAs (50 µM) were treated with miRNAs, and target concentration-dependent signal enhancements were observed. In Fig. 4a, target concentration-dependent signal increments were revealed in both fc-DNAs and afc-DNA. Also, signal enhancements of fc-DNAs and afc-DNA were shown to have similar tendencies. This similarity may be attributable to the detection efficiency of fc-DNAs, which were not affected by the afc-DNA synthesis process. For afc-DNA, both miR-21 and miR-22 were applied at various concentrations to demonstrate signal enhancement selectivity (Fig. 4b). Marina blue fluorescence showed no correlation with target concentration, while the FAM (a fluorophore detecting miR-21) and Cy3 (miR-22 detecting fluorophore) signals showed significant correlation with concentration of target miRNA. Correlation between fluorescence signal and target strand was also expressed with pseudo-color. For fluorescence expression of pseudo-colors, FAM and Cy3 matched with green and red colors, respectively. Pseudo-color codes for miR-21 and miR-22 presence are noted in Fig. 4c. For selectivity analysis against other miRNAs, five other miRNAs related to a variety of breast cancers^46,47^ (miR-1826, let-7d, miR-200c, let-7c, miR-342-3p) were selected and treated with afc-DNA at a 50 µM concentration. In Fig. 4d, only target miRNAs made significant fluorescent increments on afc-DNA. Based on this result, nanostructured DNAs were used for further cell treatment processes.

### Size-dependent signal enhancement and cellular delivery of afc-probe

**Figure 5:**
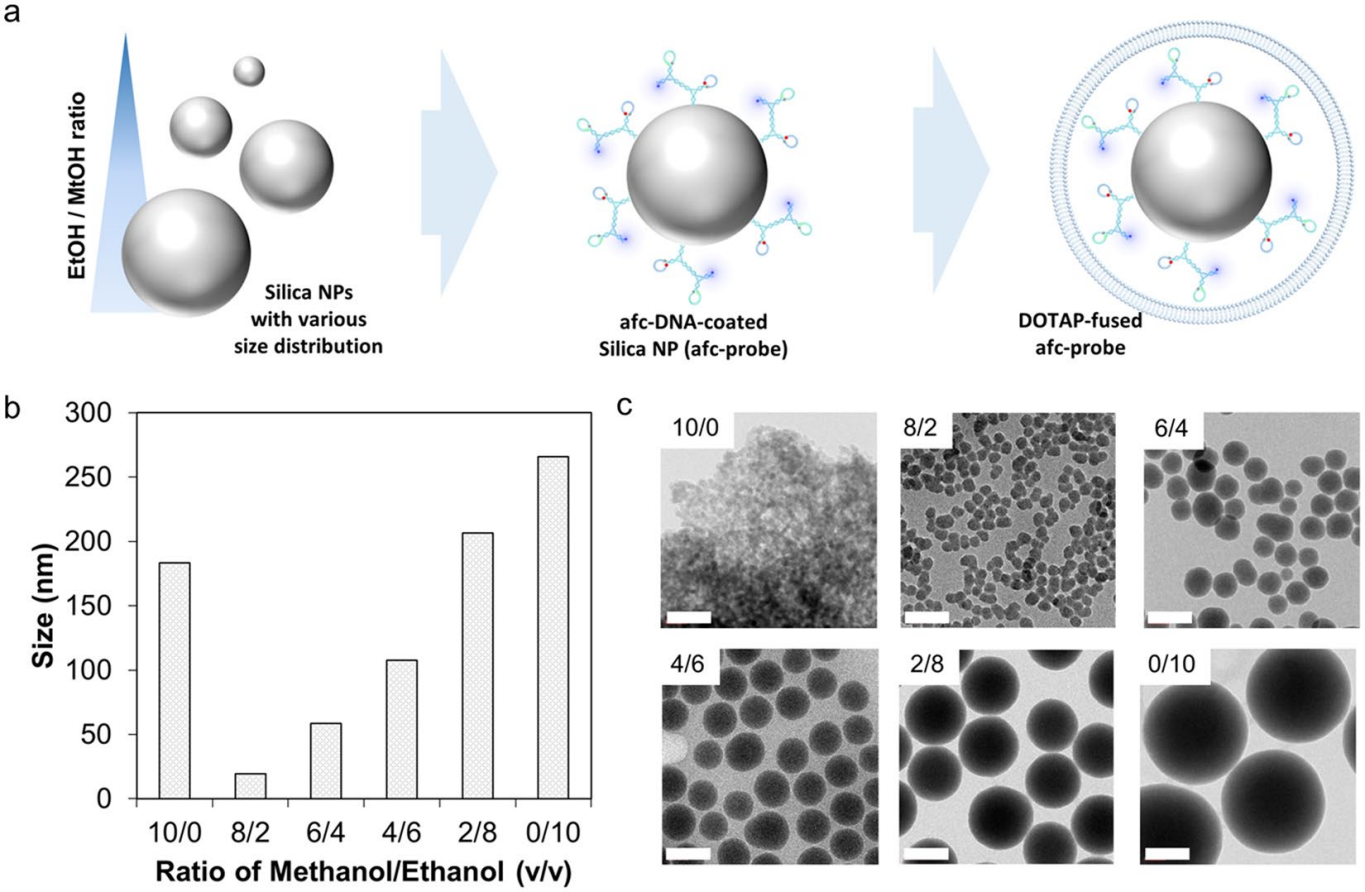
Characterization of afc-probe with various optimization parameters. (**a**) Schematic drawing of preparation procedure in DOTAP-fused afc-probe. (**b**) Size of silica nanoparticles fabricated under a variety of alcohol mixtures was measured by DLS. (**c**) Silica nanoparticles were visualized under TEM. The scale bar was 100 nm.

For signal enhancement and effective delivery of afc-DNA into the target cell, silica nanoparticles and a positively charged lipid, DOTAP were used. In previous studies, it was determined that the localized fluorescent signal on the surface of the silica particle provided substantial signal enhancement and promoted cellular internalization^48–50^. Additionally, the specific character of DOTAP for promoting internalization and endosomal escape of nanoparticles has been well explained in previous studies^51,52^. Thus, we designed the surface localization of afc-DNA on nano-sized silica as a probing system (afc-probe), and a DOTAP layer was successively fused on the top of the afc-probe (Fig. 5a).

Signal enhancement and cellular uptake according to size were verified with the silica nanoparticles sized from 20 nm to 260 nm. Silica nanoparticles with various size distributions were prepared with mixtures of ethanol and methanol. In TEM and DLS analysis, it was observed that a higher volume ratio of ethanol was associated with a larger synthesized silica size. However, when only methanol was used as a solvent, a massive aggregation of silica nanoparticles occurred, even though the size of each particle was less than 20 nm (Figs. 5b,c). Thus, silica nanoparticles ranging in size from 20 nm to 270 nm were used for further experiments. To identify the enhanced signal, fc-DNA containing only standard fluorophore was attached onto the surface of the silica nanoparticle to produce the fc-probe. Conjugation chemistry is noted in Supplementary Fig. 3a. Conjugated fc-DNA was also identified under fluorescent microscopy. The micro-scaled silica particle was used for fc-DNA conjugation and for visualization. In Supplementary Fig. 3b, the FAM of the fc-DNA was located on the surface of the silica particle. In Supplementary Fig. 3c, the amounts of conjugated fc-DNAs increased according to the size decrement of silica nanoparticles. Meanwhile, the fluorescence signal of fc-probes peaked at a 4/6 volumetric ratio of methanol and ethanol.

**Figure 6:**
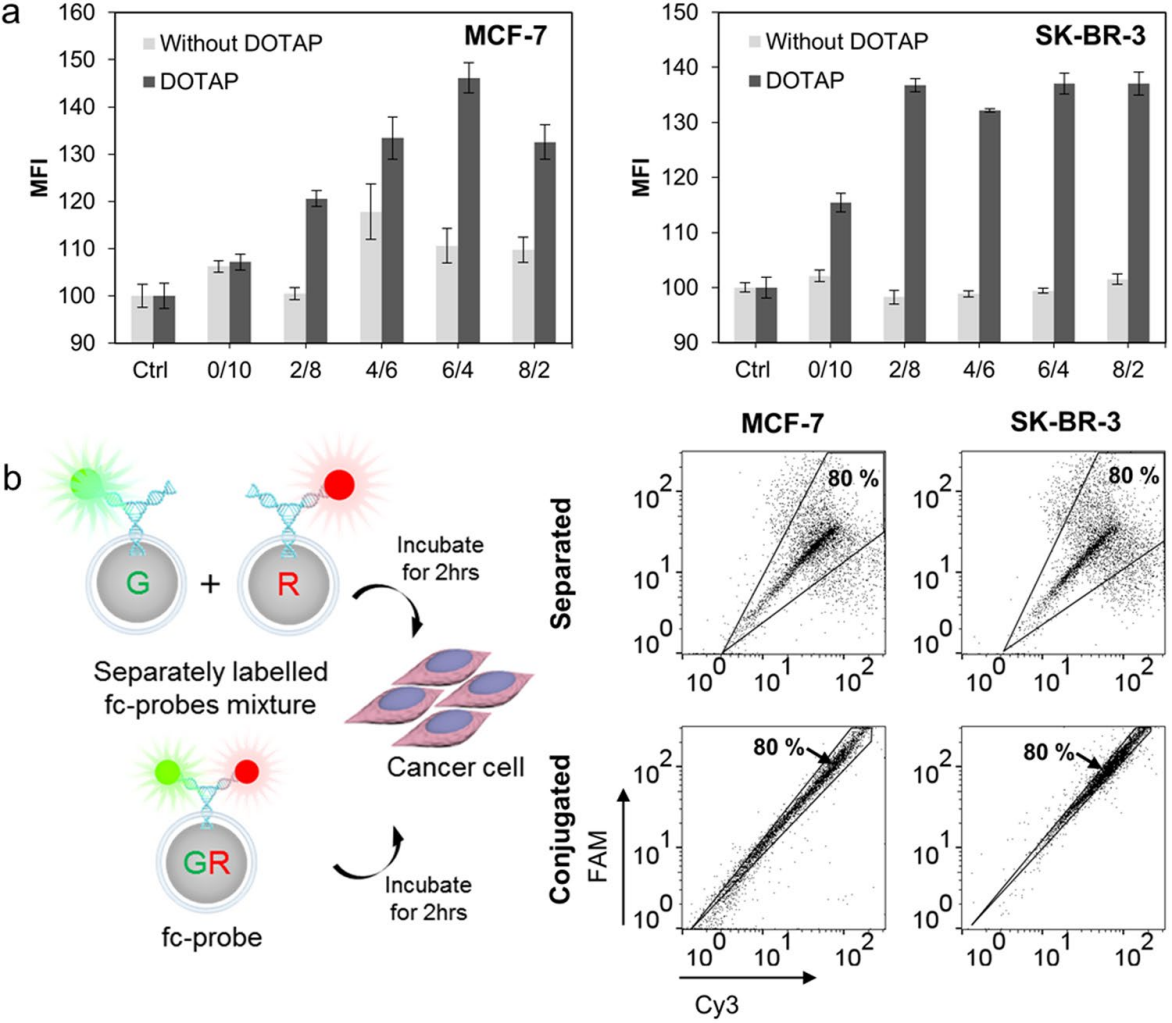
Cellular delivery of the fc-probe. (**a**) Cellular uptake efficiency of the fc-probe with or without the DOTAP fusion layer was compared with MCF-7 and SK-BR-3. (**b**) Schematic drawing of the DOTAP-fused fc-probes and its controls. The effect of nanostructured DNA in a narrow distribution of cellular uptake was measured by flow cytometry. Eighty percent of the total cell population was included within the gated black line.

Cellular delivery of the fc-probe with or without DOTAP fusion was evaluated with MCF-7 and SK-BR-3. Because the size of the DOTAP liposome used for fusion was already ~26 nm, the final diameter of DOTAP-fused fc-probes ranged from 50 nm to 270 nm (Supplementary Fig. 3d). Zeta potential before and after DOTAP treatment was measured, and the surface charge was changed from negative to positive, as expected (Supplementary Fig. 3e). In Fig. 6a, the MFI of fc-probe-treated cells was plotted by the difference of DOTAP fusion existence. In general, the DOTAP fusion layer increased the amount of internalized fc-probe. In addition, there were significant differences in internalized fc-probe based on size. A clear MFI peak was observed when the silica nanoparticles were synthesized with a 6/4 ratio of alcohol mixture in MCF-7, and similar uptake efficiency was shown in SK-BR-3 except the silica nanoparticles were synthesized at a 0/10 ratio of alcohol mixture. This phenomenon also indicated the variation of cell characteristics on particle delivery. Silica nanoparticles with a 6/4 ratio of alcohol mixture (58 nm in size) was used for miRNA diagnosis in living cells.

### Discrimination of breast cancer cell lines using miRNA signatures

To assess the self-assembly of nanostructured DNA and intracellular diagnosis, fluorescence distributions of fc-probe-treated MCF-7 and SK-BR-3 were measured with flow cytometry. FAM and Cy3 fluorophore-labelled fc-probes were used for comparison. In Fig. 6b and Supplementary Fig. 4, when the fluorophores assembled in a single nanostructure, the DNA showed narrow distribution in flow cytometry. This revealed another important reason for using nanostructured DNA for multiplexed detection in single cell level.

**Figure 7:**
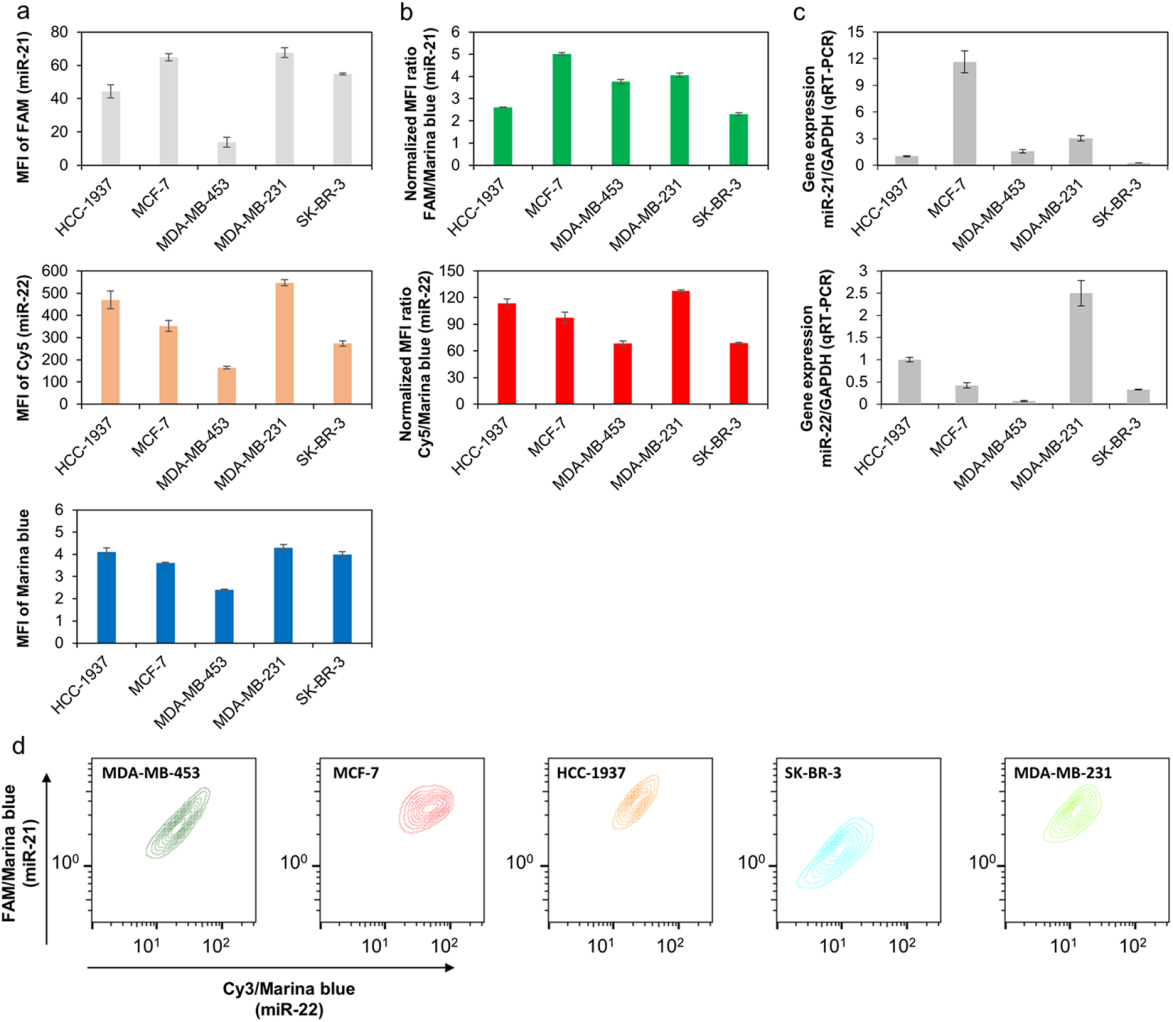
*In situ* miRNA detection in five different breast cancer cell lines. (**a**) MFIs of FAM, Cy5, and Marina Blue in each cell line as measured by flow cytometry. (**b**) MFIs of FAM and Cy5 in each cell line after normalization to the Marina Blue MFI. (**c**) Reliability of the DOTAP-fused afc-probe. The reliability was tested by comparing the miR-21 and miR-22 diagnoses via qRT-PCR. (**d**) miR-21 and miR-22 distributions in each cell line. Data were acquired via flow cytometry.

Next, five different breast cancer cell lines were selected for multiplexed diagnosis of miRNAs: HCC-1937, MCF-7, MDA-MB-453, MDA-MB-231, and SK-BR-3. The fluorescence signals of FAM (miR-21 detection) and Cy3 (miR-22 detection) were analyzed by flow cytometry. The reliability of the afc-probe was confirmed by comparative quantification of miRNAs by qRT-PCR. The concentrations of miR-21 and miR-22 were determined by calculating the normalized MFI ratio, which was derived by dividing the fluorescence intensity of the miRNA detection fluorophore (FAM or Cy3) by the standard fluorophore intensity (Marina Blue). The MFIs of the three fluorophores are shown in Fig. 7a. The FAM and Cy3 MFIs normalized to the Marina Blue MFI are displayed in Fig. 7b. These MFI results were validated by qRT-PCR (Fig. 7c). The MFI of Marina Blue was much lower in the MDA-MB-453 cells compared to all other cell lines. Thus, the FAM signal of MDA-MB-453, which indicates the concentration of miR-21, was lower than the FAM intensities of HCC-1937 and SK-BR-3, even though the miR-21 concentration was higher. However, these inaccurate results were corrected by data normalization. The order of the MFI ratios matched the quantification results from qRT-PCR. The distributions of individual cells from each line are plotted in Fig. 7d. These distributions were obtained from conventional indirect analysis. The x-axis and y-axis of the plot were obtained by dividing the detected fluorescence by the standard fluorescence of each single cell event.

## Discussion

When the importance of multiplexed, *in situ* detection of miRNAs at the single cell level became known, several techniques were developed to fulfill clinical needs. However, given the different cellular delivery kinetics of the detection system, it was hard to obtain a quantitative comparison between the different cell types. In practical diagnostic conditions, target-induced signals should be compared with a standard signal for precise quantification of the target. For qRT-PCR, housekeeping genes are typically used to standardize quantification. Using this approach, we attached an additional standard fluorophore to a molecular beacon structure for the total molecular beacon quantification system. By measuring the fluorescent signal of the standard fluorophore, it was possible to infer the amount of the diagnostic system in individual cells; the fluorescence from the molecular beacon was then compared to the standard signal, quantitatively determining the value of the miRNA concentration.

In this regard, the fluorophore arrangement is very important. FRET is the most basic on/off signal mechanism, regardless of the type of fluorophore. Thereby, the physical isolation of fluorescence from the standard signal to a molecular beacon is important, along with the fine assembly of moieties at the molecular level. To accomplish this, we used DNA nanotechnology as a molecular platform. Nanostructured DNA utilizes precise self-assembly of functional moieties and maintains the three-dimensional structure following the sequence design. To quantify multiple miRNA diagnoses, fc-DNA and afc-DNA containing standard fluorophores and molecular beacons were fabricated. fc1-DNA contained standard fluorophore and the miR-22 targeting-molecular beacon, while fc2-DNA contained the miR-21 targeting-molecular beacon.

First, the assemblies and conformation of nanostructured DNA were investigated using gel electrophoresis and molecular dynamics simulations. Hybridization of three oligonucleotide strands for fc-DNAs and T4-ligase crosslinking of fc1- and fc2-DNA induced a shift in band locations compared to single oligos, mostly a result of the higher molecular weights and conformational topology. In the molecular dynamics simulation program, oxDNA, the assembly and thermodynamic stability of the nanostructured DNA were verified. The hydrogen bonding energy and the three-dimensional structures of each nanostructured DNA were maintained throughout simulation. The distances among the fluorophores were also maintained.

With regards to target detection, FAM and Cy3 fluorophore-containing molecular beacons were used to detect miR-21 and miR-22, respectively, and Marina blue was used as a standard fluorophore. Both fc-DNAs and afc-DNA showed target concentration-dependent signal enhancements without the interference of other miRNAs.

For the efficient detection of target miRNAs in living cells, afc-DNA was coated on the silica nanoparticle and further fused with a DOTAP lipid layer. Localization of nanostructured DNAs on the silica nanoparticle enhanced the fluorescent signal intensity, and the DOTAP fusion layer was designed to promote cellular uptake and endosomal escape. Both signal enhancement and cellular uptake were optimized by the size of the silica nanoparticle. It was observed that when the size of the nanoparticle was around 70 nm in diameter, the highest amount of fluorescent signal outcome in both MCF-7 and SK-BR-3 cell lines. Additionally, when the fluorophores assembled in a single nanostructure, fluorescent signals showed a narrow distribution for the flow cytometry. This difference demonstrated one of the reasons for using a nanostructured DNA-based system for multiplexed diagnosis. Misunderstanding is possible when detection systems are applied to cells in a biased manner. This possibility can be drastically reduced with nanostructured DNA because it allows for precise assembly of functional moieties at the molecular level.

The target diagnosis reliability of the afc-probe was compared with the current gold standard technique, qRT-PCR. For both detection methodologies, the order of concentrations of miR-21 and miR-22 for five different breast cancer cell lines (HCC-1937, MCF-7, MDA-MB-453, MDA-MB-231, and SK-BR-3) agreed with each other. In addition, it was possible to obtain data on the heterogeneity in miRNAs concentration in each single cell line when the afc-probe system was used. This result indicates that this probing system can be used to detect tumor heterogeneity. The absolute value of miRNA concentrations measured with the afc-probe did not correspond with the result from the qRT-PCR. In the afc-probe, molecular beacons were used to indicate the presence of the targets. In the molecular beacon system, the fluorescent signal takes place along the linear equation even though the target strand is existed in various concentrations. Therefore, the final results among the samples could not appear exponentially. This has been one of the universal problems of target detection methods through the signal amplification^53^. To solve this problem, several techniques such as toehold-mediated strand displacement have been developed^54,55^, but further progress is needed for an intracellular diagnosis application.

In summary, we demonstrated that a novel nanostructured DNA-based probe can be used for multiplexed miRNA detection in various breast cancer cell lines. The precise assembly of nanostructured DNA enables the detection of multiplexed miRNA in living cells and reduces bias due to different cell types or characteristics. The size and surface character of the afc-probe were also optimized for efficient cellular delivery and its reliability was confirmed by comparison with commercialized qRT-PCR analysis. The flow cytometry results showed that it is possible to directly compare miRNA expression levels between different breast cancer cell lines having specific characteristics. The straightforward application of intracellular analysis methods enables the realistic and concrete design of intracellular diagnostic systems. We anticipate that this methodology could be used for precise and easy diagnosis of tumor heterogeneity in clinical applications.

## Materials and Methods

### Nanostructured DNA material preparation

DNA nanostructure was prepared as described by previous reports^56^. For concise hybridization, the annealing process was performed in a thermocycler provided by the Mastercycler Pro of Eppendorf (Westbury, NY, USA). After heating at 95 °C for 2 minutes, stepwise temperature was decreased from 60 °C to 20 °C at 1 °C per minute. afc-DNA was obtained by enzymatic ligation of two different fc-DNAs containing complementary four-base sticky ends. Equimolar fc-DNAs were mixed with a 3 Weiss unit of T4 ligase and ligase buffer. The reaction was incubated at 4 °C for 16 hours. After fc-DNA and afc-DNA preparation, the solution buffer was changed to distilled water.

### Coarse-grain model for simulation of nanostructured DNA behaviour

For three-dimensional configuration of nanostructured DNA, an oxDNA coarse-grain simulation program was used. The version of software used in this study was oxDNA2, and the code for simulation was available as a free-download. Detailed input values and experimental conditions are noted in Supplementary Section 1.

### Preparation of silica core nanoparticles with various sizes

Silica nanoparticles with size variation were synthesized following a previous report^57^. A mixed solution of both ethanol and methanol was used as a media of silica nanoparticle fabrication, and the volumetric ratio between ethanol and methanol was varied to adjust the size of the silica nanoparticle.

### Preparation of the afc-probe

The afc-probe was simply prepared by chemical conjugation of nanostructured DNA onto silica nanoparticles and covered with fused DOTAP from Avanti Polar Lipids, Inc. (Alabaster, AL, USA). The procedure has been described in a previous report^58^ with minor modifications. First, surface amine-modification of the silica nanoparticle was achieved by adding APTMS from Sigma-Aldrich Co. (St. Louis, MO, USA) to silica nanoparticle. Cyanuric chloride was added dropwise. After 2 hours of incubation with gentle stirring, the cyanuric chloride-modified silica nanoparticles were achieved. The silica nanoparticles were reacted with amine-modified nanostructured DNA. For DOTAP coating, former protocols was referenced^59^. A tip sonication (10% amplitude for 10 min) by Q700 provided from Qsonica, LLC. (Newtown, CT, USA) on ice was performed to create unilamellar DOTAP liposome. Immediately after sonication, DNA nanostructure-conjugated silica nanoparticles were then mixed with DOTAP solution for fusion. After one hour of incubation, the synthesized afc-probe was washed thoroughly with distilled water three times to remove excessive DOTAPs and stored at 4 °C until use.

### afc-probe treatment and fluorescent signal measurement

For *in situ* diagnosis, breast cancer cell lines were used. MCF-7, SK-BR-3, MDA-MB-231, MDA-MB-453, and HCC-1937 were purchased from the Korean Cell Line Bank (KCLB). The afc-probe was applied to each cell line for 2 hours. The afc-probe-treated cells were harvested and washed with PBS three times to remove the remaining free afc-DNA probe. Stepwise thermal decrement (−1 °C/min) from 37 °C to 4 °C was used to treat the cells in a PCR Mastercycler® pro from Eppendorf (Westbury, NY, USA). Flow cytometry with a MACSQuant VYB from Miltenyi Biotec (Auburn, CA, USA) was used to measure the fluorescent signals.

### Quantitative real-time polymerase chain reaction (qRT-PCT)

qRT-PCR analysis was performed according to our previous protocol^59^. TaqMan Fast Universal PCR Master Mix from Applied Biosystems (Foster City, CA, USA) was used for the reaction. The comparative threshold cycle (Ct) method was used to measure the relative expression of each target by normalizing the gene expression of the target gene to that of an endogenous reference transcript, GAPDH.

## Electronic supplementary material

Supplementary information
