# Supplementary material for: Fluorescence-coded DNA Nanostructure Probe System to Enable Discrimination of Tumor Heterogeneity via a Screening of Dual Intracellular microRNA Signatures in situ
Source: Sci Rep. 2017 Oct 18;7:13499. doi: 10.1038/s41598-017-13456-3 (PMC5647416; doi:10.1038/s41598-017-13456-3)
Supplement: Supplementary file 1 — Supplementary information [file 41598_2017_13456_MOESM1_ESM.doc]

Supplementary Information for

**Fluorescence-coded DNA Nanostructure Probe System to Enable Discrimination of Tumor Heterogeneity via a Screening of Dual Intracellular microRNA Signatures in situ**

Seung Won Shin, Byoung Sang Lee, Kisuk Yang, Lunjakorn Amornkitbamrung, Min Su Jang, Bo Mi Ku, Seung-Woo Cho, Jung Heon Lee, Hojae Bae, Byung-Keun Oh, Myung-Ju Ahn, Yong Taik Lim & Soong Ho Um*

**Contents**

**Supplementary Section and Figures**

**Methods** Detailed experimental methods

**Section 1** oxDNA program simulation parameters

**Figure S1** Detailed sequence of nanostructured DNAs and their corresponding gel electrophoresis images

**Figure S2** Target miRNA detection kinetics of fc-DNA.

**Figure S3** DNA conjugation onto silica and characterization of intermediates in each step

**Figure S4** *In situ* fc-probe analysis

**Figure S5** Full-length gel electrophoresis images

**Methods**

**Nanostructured DNA material preparation.** The Marina blue modified oligonucleotide was synthesized from Bioneer Co. Ltd. (Daejeon, South Korea). All other oligonucleotides that contained fc-DNAs and afc-DNA and target miRNAs were purchased from Integrated DNA Technologies, Inc. (Coralville, IA, USA). T4 ligase and ligase buffer were purchased from Promega Corp. (Madison, WI, USA). Lyophilized DNA oligonucleotides composing fc-DNAs and afc-DNA were dissolved in TE buffer (10 mM Tris, pH 8.0, 0.1 mM EDTA) to 100 μM concentration. Each fc-DNA was obtained by hybridization of three oligonucleotide strands. The oligonucleotides were mixed at an equimolar ratio for the annealing process. For concise hybridization, the annealing process was performed in a thermocycler provided by the Mastercycler Pro of Eppendorf (Westbury, NY, USA). After heating at 95 ^o^C for 2 minutes, stepwise temperature was decreased from 60 ^o^C to 20 ^o^C at 1 ^o^C per minute. afc-DNA was obtained by enzymatic ligation of two different fc-DNAs containing complementary four-base sticky ends. Equimolar fc-DNAs were mixed with a 3 Weiss unit of T4 ligase and ligase buffer (300 mM Tris-HCl, pH 7.8, 100 mM MgCl_2_, 100 mM dithiothreitol (DTT), and 10 mM ATP). The reaction was incubated at 4 ^o^C for 16 hours. After fc-DNA and afc-DNA preparation, the solution buffer was changed to distilled water by sequential filtration using an Amicon 3K centrifugal filter from Millipore Inc. (Bedford, MA, USA), and the final concentration was adjusted to 100 μM. The samples were stored at 4 ^o^C until use.

**Coarse-grain model for simulation of nanostructured DNA behavior.** For three-dimensional configuration of nanostructured DNA, an oxDNA coarse-grain simulation program was used. The version of software used in this study was oxDNA2, and the code for simulation was available as a free-download. For simulation, the same oligonucleotide strands used in the synthesis of fc-DNAs and afc-DNA were utilized to obtain the structure variation and parameters for interaction of nanostructured DNA.

**Preparation of silica core nanoparticles with various sizes.** A mixed solution of both ethanol and methanol was used as a media of silica nanoparticle fabrication, and the volumetric ratio between ethanol and methanol was varied to adjust the size of the silica nanoparticle. In brief, 46 ml of mixed alcohols, 1 ml of distilled water, and 3 ml of NH_4_OH (28.0~30.0 %) from Sigma-Aldrich Co. (St. Louis, MO, USA) were added and gently stirred for 10 minutes. Then, 0.6 ml of tetraethyl orthosilicate (TEOS) from Sigma-Aldrich Co. (St. Louis, MO, USA) was added dropwise. The reaction mixture was incubated for 16 hours at room temperature with gentle stirring. Synthesized silica nanoparticles were washed in pure ethanol, followed by three rounds of centrifugation (15,000 rpm, 30 minutes). The size and zeta potential were measured by high-resolution transmission electron microscopy (HR-TEM) and dynamic light scattering (DLS). For HR-TEM, JEM-3010 from JEOL, Ltd. (Tokyo, Japan) was used, and Zetasizer Nano ZS from Malvern Instruments, Ltd. (Worcestershire, UK) was used for DLS. For fluorescence microscopic visualization, the micro-scaled silica particles were fabricated based on the procedures in a previous report.(45) The total reaction mixture containing 1.5 mg of KCl in 38 ml of ethanol, 6 ml of distilled water, and 3 ml of NH_4_OH was then added and gently stirred for 10 minutes. Upon reaction, 35 ml of ethanol, which dissolves 6.04 g of TEOS, was treated at a supply rate of 0.12 ml/min. The reaction was incubated for 16 hours under gentle stirring, and the micro-scaled silica particles were obtained. The silica particles were thoroughly washed with pure ethanol three times, followed by centrifugation.

**Preparation of the afc-probe.** The afc-probe was simply prepared by chemical conjugation of nanostructured DNA onto silica nanoparticles and covered with fused DOTAP from Avanti Polar Lipids, Inc. (Alabaster, AL, USA). First, surface amine-modification of the silica nanoparticle was achieved by adding 0.8 ml of APTMS from Sigma-Aldrich Co. (St. Louis, MO, USA) to 20 mg of silica nanoparticle in a reaction buffer (a mixture of 38 ml of ethanol, 2 ml of distilled water, 0.25 ml of acetic acid) and then stirred for 2 hours at room temperature. The amine-modified silica nanoparticles were washed twice with pure ethanol and then three times in acetonitrile from Sigma-Aldrich Co. (St. Louis, MO, USA). The final volume of silica nanoparticles in acetonitrile was adjusted to 3 ml, and 115.2 mg of cyanuric chloride from Sigma-Aldrich Co. (St. Louis, MO, USA) in 1 ml of acetonitrile was added dropwise. After 2 hours of incubation with gentle stirring, the cyanuric chloride-modified silica nanoparticles were washed twice with acetonitrile, three times with ethanol, and three times with borate buffer (pH 8.5). In borate buffer conditions, the silica nanoparticles were reacted with amine-modified nanostructured DNA. For the reaction, 4 mg of cyanuric chloride-modified silica nanoparticles were mixed with 25 μl of prepared nanostructured DNA for 16 hours at room temperature and vortexed gently. After conjugation, the remaining nanostructured DNA was removed by centrifugation and then washed three times with distilled water. Synthesized afc-probe was stored at 4 ^o^C until use. To remove the remaining chloroform, 3.5 mg of DOTAP solution was incubated under vacuum condition for 16 hours and rehydrated in 1.4 ml of distilled water. A tip sonication (10% amplitude for 10 min) by Q700 provided from Qsonica, LLC. (Newtown, CT, USA) on ice was performed to create unilamellar DOTAP liposome. Immediately after sonication, 4 mg of DNA nanostructure-conjugated silica nanoparticles were then mixed with DOTAP solution for fusion. After one hour of incubation, the synthesized afc-probe was washed thoroughly with distilled water three times to remove excessive DOTAPs and stored at 4 ^o^C until use.

**afc-probe treatment and fluorescent signal measurement.** For in situ diagnosis, breast cancer cell lines were used. MCF-7, SK-BR-3, MDA-MB-231, MDA-MB-453, and HCC-1937 were purchased from the Korean Cell Line Bank (KCLB). After seeding each cell line in a 24-well plate (1 × 10^5^ cells/well), cells were cultured to reach 80 % confluency. Original media was removed and the bound cells were washed with PBS, the afc-probe (500 μg of silica) was applied to each cell line for 2 hours. The afc-probe-treated cells were harvested and washed with PBS three times to remove the remaining free afc-DNA probe. Stepwise thermal decrement (-1 ^o^C/min) from 37 ^o^C to 4 ^o^C was used to treat the cells in a PCR Mastercycler® pro from Eppendorf (Westbury, NY, USA). Flow cytometry with a MACSQuant VYB from Miltenyi Biotec (Auburn, CA, USA) was used to measure the fluorescent signals.

**Quantitative real-time polymerase chain reaction (qRT-PCT).** TaqMan Fast Universal PCR Master Mix from Applied Biosystems (Foster City, CA, USA) was used for the reaction. The gene expression profiles of the cells were measured using TaqMan Gene Expression Assays from Applied Biosystems (Foster City, CA, USA) for each target miR-21; (Hs04231424_s1), miR-22; (Hs00993773_g1) and glyceraldehyde 3-phosphate dehydrogenase (GAPDH); (Hs02758991_g1). The comparative threshold cycle (Ct) method was used to measure the relative expression of each target by normalizing the gene expression of the target gene to that of an endogenous reference transcript, GAPDH.

***Section 1: oxDNA program simulation parameters***

Structural analysis of fc-DNAs and afc-DNA in an oxDNA program was achieved by two successive steps; 1) an initial structure generation step and 2) a structure analysis step. A “mutual trap” function was used to fabricate the basic structure from the fc-DNAs and afc-DNA, which was provided in the oxDNA program. Mutual trap function forces two other bases into close proximity and is generally used for initial DNA hybridization. In our cases, the second bases from each end of the oligonucleotides were trapped together for rapid fabrication of the initial structure. For the afc-DNA, several additional bases were trapped together to reduce unwanted secondary structures. Trapped base pairs are noted below for better visual comprehension (red box).


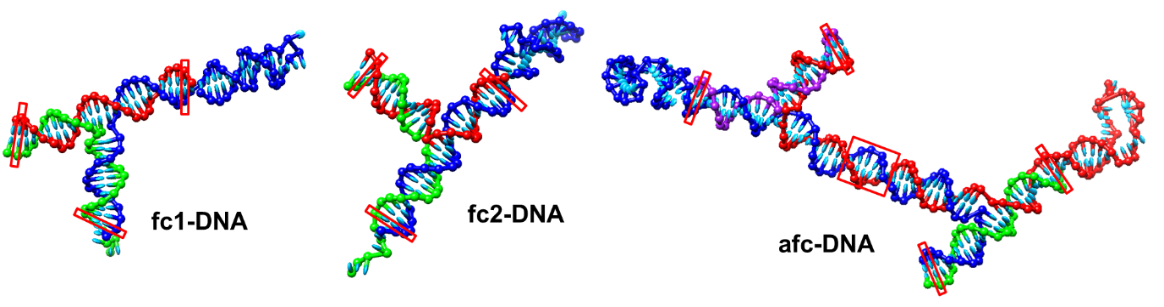


After the initial parameter was generated as expected, additional structure analysis, including hydrogen bonding energy extraction, and the distance of each end of the arms were determined by sequence-dependent molecular dynamic (MD) simulation without mutual trapping. The input parameter for initial structure generation and structure analysis are noted below. Additional settings used default values.

|  | **Simulation type** | **Backend** | **Interaction type** | **Salt concentration** | **Simulation steps** |
| --- | --- | --- | --- | --- | --- |
| **Initial**  **structure generation** | Virtual move Monte Carlo  (VMMC) | CPU | DNA2 | 0.15 | 1.00E+05 |
| **Structure**  **analysis** | Molecular dynamics (MD) | CUDA | DNA2 | 0.15 | 1.00E+07 |
|  | **Temperature**  **(^o^C)** | **Average sequence file** | **Used sequence dependent file** | **Simulation data print out**  **Interval (Step)** | **External force (Mutual trap)** |
| **Initial**  **structure generation** | 37 | Not available | Not available | 100 | On |
| **Structure**  **analysis** | 37 | Off | oxDNA2_sequence_dependent_parameters.txt | 100 | Off |

**Figure S1.** (a) Detailed sequence information for both fc-DNA and afc-DNA. afc-DNA was created using T4 enzymatic ligation of two fc-DNAs via four-base sticky end hybridization. (b) Gel electrophoresis image of fc-DNAs and afc-DNA. The gel image including fc1-a, fc1-a/fc1-b and fc1-DNA was cropped from single part of gel. Also, the gel image including fc1-DNA, fc2-DNA and afc-DNA was cropped from single part of gel. A 25 bp DNA ladder was used for comparison. The ladder bands ranged from 25 bp to 300 bp, in 25 bp increments. The full-length gel images were provided in Figure S5.

**Figure S2.** Target miRNA detection kinetics of fc-DNA. (a) Normalized mean fluorescence intensity (MFI) of fc-DNA incubated with various concentrations of target miRNA under 37^o^C. (b) MFI of fc-DNA with target miRNAs before annealing (square) and after annealing (circle).


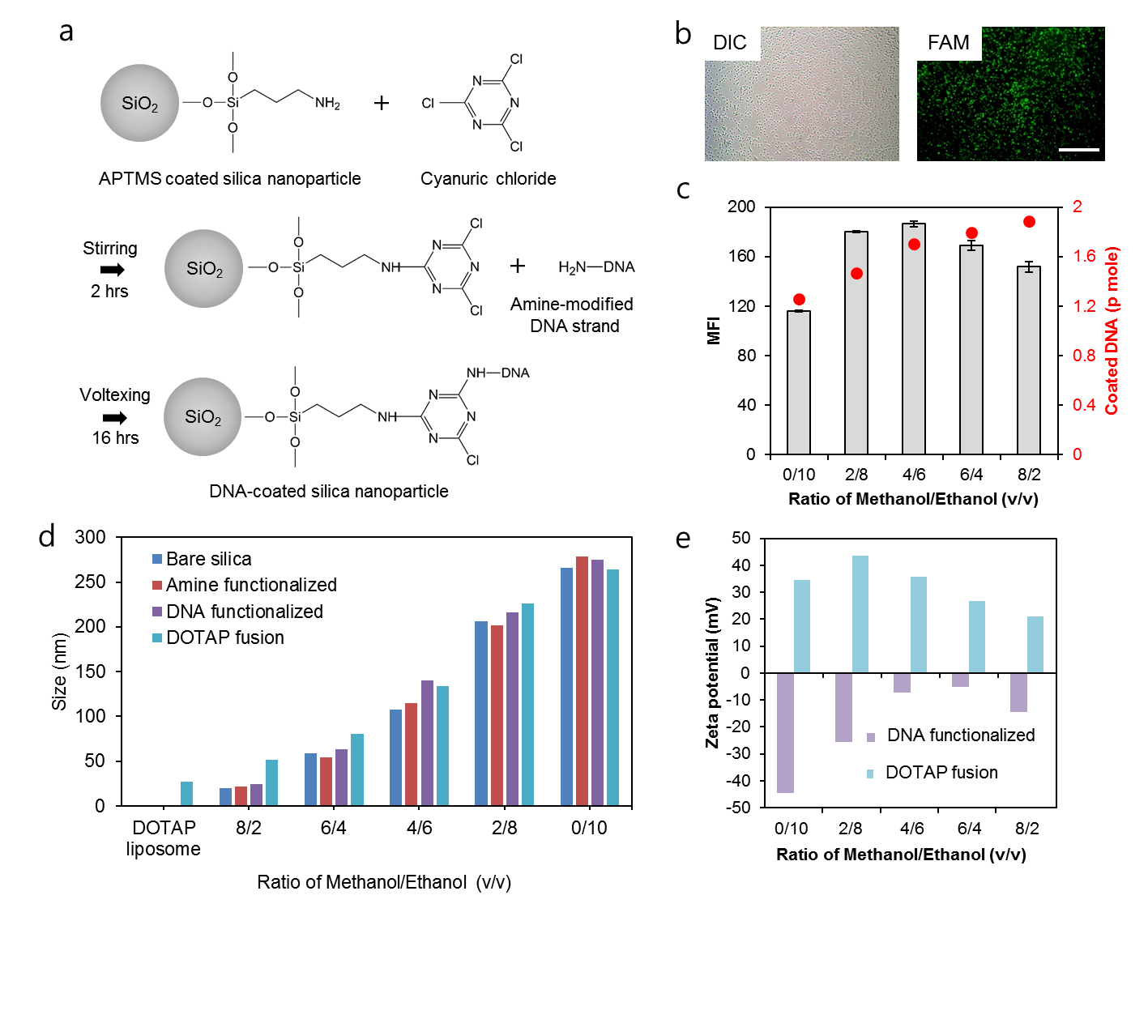


**Figure S3.** DNA conjugation onto silica and characterization of intermediates in each step. (a) Nanostructured DNA was conjugated via a simple conjugation method among two primary amine derivatives in each precursor (i.e., Aminopropyl trimethoxy-silane (APTMS)-coated silica and oligos) with the help of a cyanuric chloride linker. (b) Fluorescent microscopic image of fc-DNA-coated micro-scaled silica particles. DIC simply indicated differential interference contrast. The scale bar was 50 µm. (c) Signal enhancement of fc-DNA by localization on the silica nanoparticles was tested by comparing the MFI of fc-probe (bar graph) and the amount of conjugated fc-DNA calculated from the MFI supernatant (dot graph). (d) Size of silica nanoparticles synthesized with various volumetric ratio of alcohols (Methanol/Ethanol) was measured through a surface functionalization procedure. The size of the DOTAP liposome was measured immediately after the sonication. (e) Zeta potential of fc-probe before and after the DOTAP fusion.


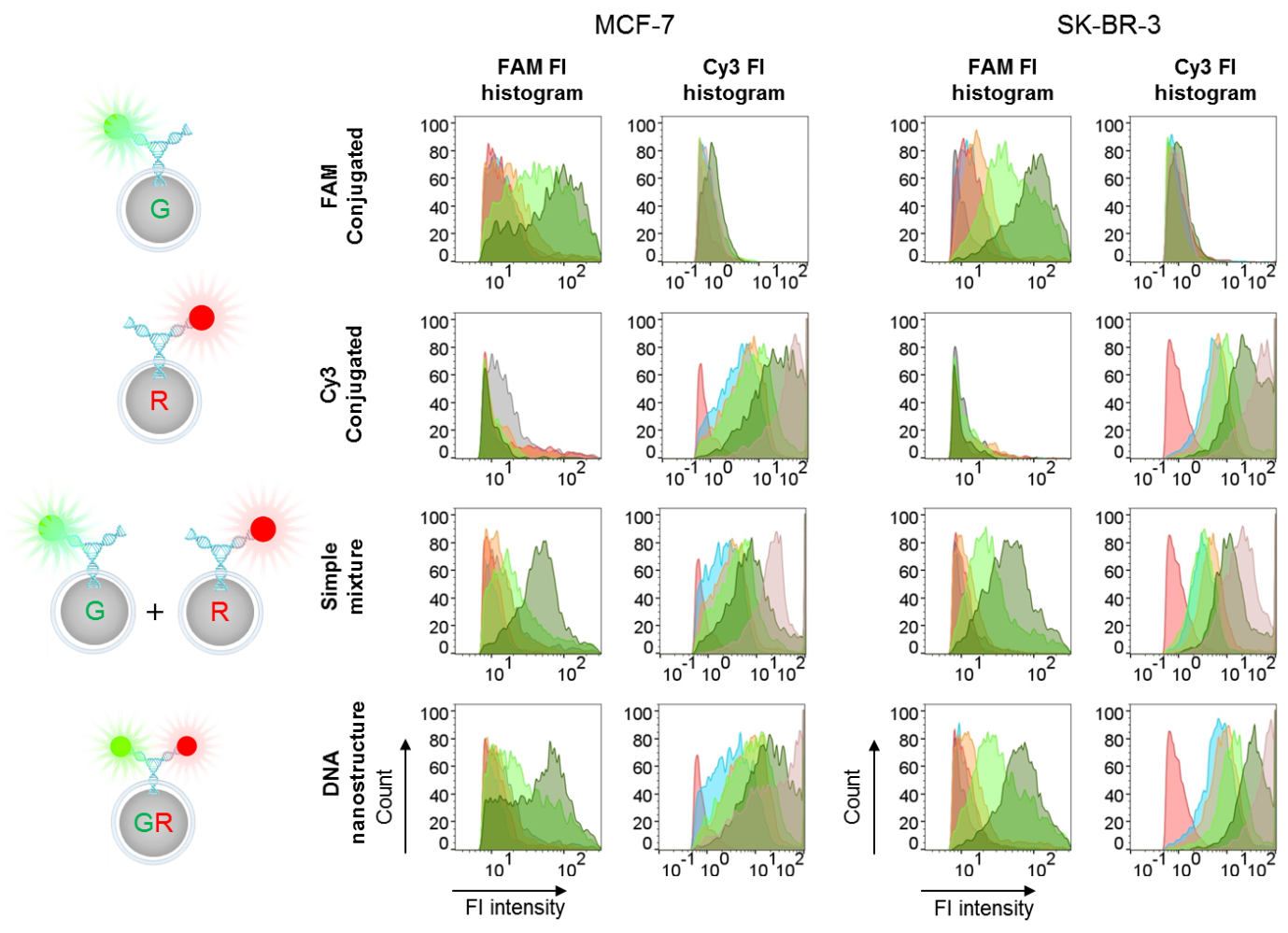


**Figure S4.** DNA nanostructure-dependent fluorescence signal increment in MCF-7 and SK-BR-3. In a histogram of FAM and Cy3, the fluorescence intensity (FI) of both MCF-7 and SK-BR-3 increased independently. Meanwhile, when a simple mixture and nanostructured DNA-conjugated fc-probe were used to treat cell lines, FI of both FAM and Cy3 increased.


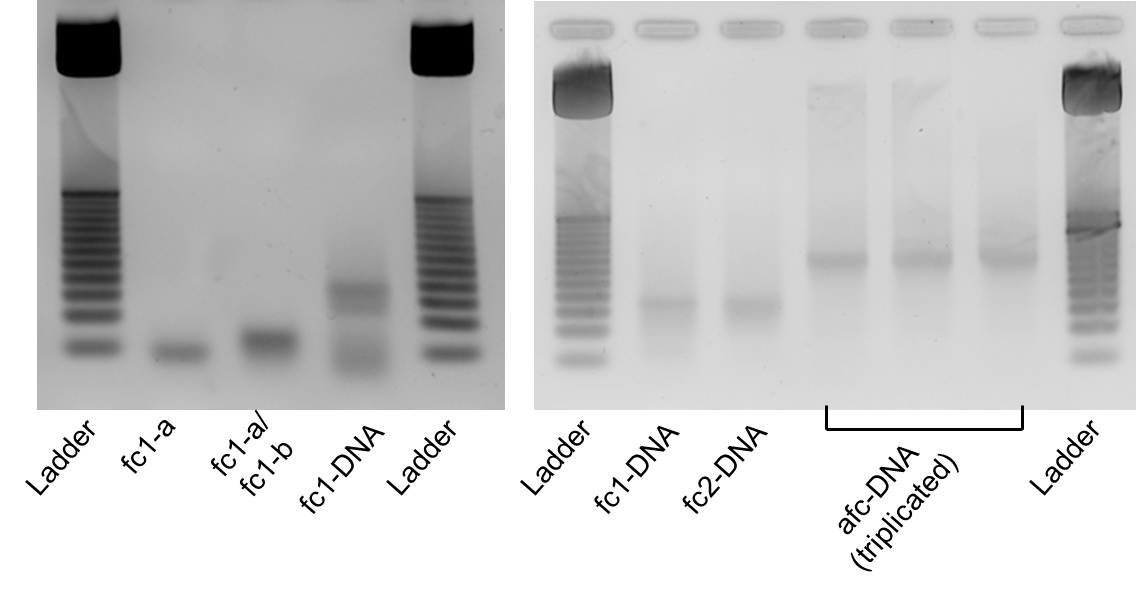


**Figure S5.** Full-length gel electrophoresis image of fc-DNAs from Figure S1b.
